# Force-Sensitive Interface Engineering in Flexible Pressure Sensors: A Review

**DOI:** 10.3390/s22072652

**Published:** 2022-03-30

**Authors:** Guojun Tai, Dapeng Wei, Min Su, Pei Li, Lei Xie, Jun Yang

**Affiliations:** 1 Chongqing Institute of Green and Intelligent Technology, Chinese Academy of Sciences, Chongqing 400714, China; taiguojun@cigit.ac.cn (G.T.); dpwei@cigit.ac.cn (D.W.); sumin@stu.cqut.edu.cn (M.S.); 20160802049@cqu.edu.cn (P.L.); 2 Chongqing School, University of Chinese Academy of Sciences, Chongqing 400714, China; 3 Department of Optoelectronic Engineering, Chongqing University, Chongqing 400044, China; lxie@cqu.edu.cn

**Keywords:** flexible pressure sensors, force-sensitive interface, interface engineering, microstructures

## Abstract

Flexible pressure sensors have received extensive attention in recent years due to their great importance in intelligent electronic devices. In order to improve the sensing performance of flexible pressure sensors, researchers are committed to making improvements in device materials, force-sensitive interfaces, and device structures. This paper focuses on the force-sensitive interface engineering of the device, which listing the main preparation methods of various force-sensitive interface microstructures and describing their respective advantages and disadvantages from the working mechanisms and practical applications of the flexible pressure sensor. What is more, the device structures of the flexible pressure sensor are investigated with the regular and irregular force-sensitive interface and accordingly the influences of different device structures on the performance are discussed. Finally, we not only summarize diverse practical applications of the existing flexible pressure sensors controlled by the force-sensitive interface but also briefly discuss some existing problems and future prospects of how to improve the device performance through the adjustment of the force-sensitive interface.

## 1. Introduction

With the progress of society and the rapid development of the industries, smart electronic devices, such as smart phones, smart homes, and smart robots, have grown rapidly. One of the important technologies that exist in integrated smart electronic devices is sensor technology. The sensor can detect external signals (pressure [1,2], temperature [3,4,5], humidity [6,7,8], gas [9,10,11], etc.) and transmit them to the corresponding receiver for device control. As an important branch of the sensor, the flexible pressure sensor can recognize the applied force from the outside, convert the mechanical signal into an electrical signal and finally output it. Therefore, it can be applied in electronic skin [12,13], flexible wearable devices [14,15], medical and health [16,17], robotics [18,19,20] and other fields, which shows extensive development and application prospects.

The flexible pressure sensor can be divided into piezoresistive type [21,22,23], pressure–capacitive type [24,25,26], piezoelectric type [27,28,29], and piezomagnetic type [30,31], according to the working principle. It is generally known that high performance is the prerequisite for flexible pressure sensors to be widely used. Although there have been many reviews to introduce various flexible pressure sensors separately or comprehensively from the types, principles, and applications of the devices [32,33,34,35], these articles do not have a systematic explain about the means of improving the performance of the device. In addition, many articles introduce the interface microstructures of the device only from the perspective of materials and micro-nano-processing technology. However, the impact of microstructures on the performance of the flexible sensor from the perspective of the force-sensitive interface is not discussed [36,37]. In this way, it is essential to explore the influence of the microstructures of the force-sensitive interface and the device structure design on the performance of the flexible pressure sensor from the perspective of the device’s force-sensitive interface.

Herein, with the focus on the impact of the force-sensitive interface on the sensor performance, a comprehensive and important review is presented from the performance of the flexible pressure sensor, interface microstructures, device structure design, and application. In order to make it clear, this article mainly focuses on two types of devices, flexible piezoresistive sensors and flexible capacitive pressure sensors. Starting from the working principle and sensing performance of the sensor, the performance indicators of flexible pressure sensors now generally recognized and the existing performance control methods (material improvement and force-sensitive interface micro-nanostructure design) are introduced first. Subsequently, the types of force-sensitive interfaces with introduced micro-nanostructures are classified, which the existing types of force-sensitive interfaces are divided into periodic/quasi-periodic force-sensitive interfaces and irregular force-sensitive interfaces. Afterwards, the control strategy of the device is combed from the overall structure of the device with the influence of different device structures on the performance of the sensor is explored. Then, the potential applications of flexible pressure sensors regulated by force-sensitive interfaces are concluded from the aspects of human health detection, intelligent robots, and metaverse. Finally, the research on the adjustment of the force-sensitive interface to the flexible pressure sensor is summarized; additionally, some potential problems and prospects for the future are also put forward.

## 2. Fundamental Designs of Flexible Pressure Sensors

### 2.1. Pressure Trasduction Sensing Mechaninsm

As a flexible electronic component that can detect external forces, the basic principle of it is to convert the mechanical quantity applied to the sensor from the outside into an electrical quantity.

**Figure 1:**
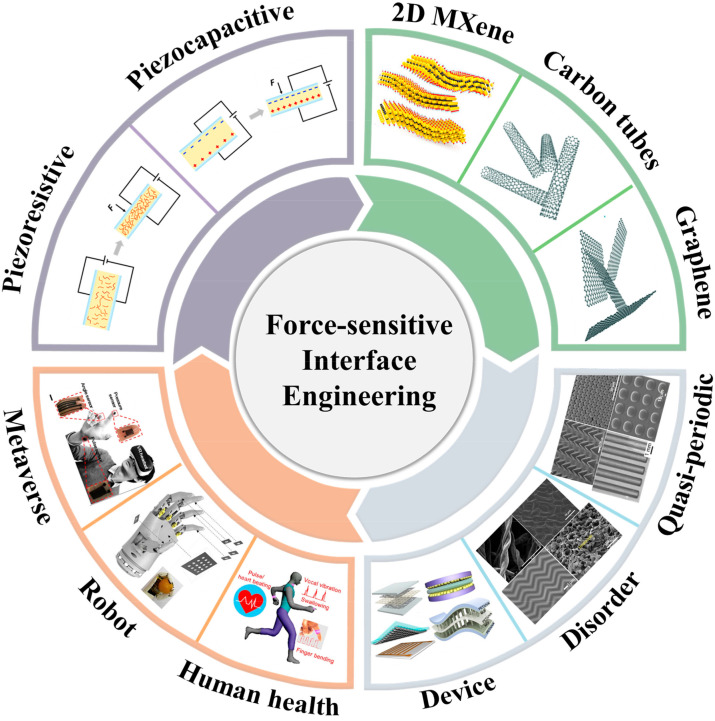
A diagram about principle, material, device structure, and application of the flexible pressure sensor obtained through the adjustment of the force-sensitive interface. Images adapted from: mechanisms [86] (Copyright 2019, Royal Society of Chemistry), materials (2D Mxene [87] (Copyright © 2020, American Chemical Society)), Carbon tubes/Graphene, [88] (Copyright 2020, RSC Pub), structures (quasi-periodic, [70,80,89,90] (Copyright © 2018 Elsevier Ltd. Copyright © 2019 WILEY-VCH Verlag GmbH & Co., KGaA, Weinheim, Germany. Copyright © 2019, American Chemical Society. Copyright © 2018 WILEY-VCH Verlag GmbH & Co., KGaA, Weinheim), disorder, [38,39,82,91] (Copyright © 2020, the author(s). Copyright © 2021, American Chemical Society. Copyright © 2014 WILEY-VCH Verlag GmbH & Co., KGaA, Weinheim. Copyright 2015, Royal Society of Chemistry), device [39,54,73,89] (Copyright © 2021, American Chemical Society. Copyright © 2017 WILEY-VCH Verlag GmbH & Co., KGaA, Weinheim. Copyright © 2020 Elsevier Ltd. Copyright © 2019, American Chemical Society), applications (human health, [92] (Copyright © 2020 Elsevier Ltd.), robot [93] (Copyright © 2019 Elsevier Ltd.), and mataversr [94] (Copyright © 2018 Elsevier B.V.)).

Flexible capacitive pressure sensor is composed of electrodes and dielectrics. The “sandwich” structure of “electrode–dielectric–electrode” is commonly used in device structure design. Under the action of external pressure, the deformation of the sensor device also changes the dielectric constant of the device; that is, changing the *d* and ε in Equation (1) [33]:

(1) $$C=\frac{\varepsilon S}{4\pi kd}$$

where *C* is the capacitance, ε is the dielectric constant, *S* is the effective area of the electrode, *k* is the Coulomb constant, and *d* is the distance between the electrode plates, thus resulting in the capacitance change of the flexible capacitive pressure sensor (as illustrated in Figure 1). There is a special kind of sensor among the flexible capacitive pressure sensor called ionic flexible capacitive pressure sensor. Different from usual flexible capacitive pressure sensors, the working principle of this type of device is that the external force brings the device deformation, which affects the contact area between the electrode and the active dielectric layer, thereby causing the capacitance change of the device [38]. In addition, the flexible capacitive pressure sensor has the advantages of low output energy and good dynamic response. However, the high difficulty of capacitive signal acquisition restricts its application seriously.

Flexible piezoresistive sensors usually contain electrodes and active conductive materials. Under external force stimulation, the resistance of the piezoresistive sensor changes, so that the output electrical signal of the device changes correspondingly (as illustrated in Figure 1). The flexible piezoresistive sensor includes two types of devices. One is the contact resistance type, which the output electrical signal change depends on the contact resistance change of the two conductive active materials of the piezoresistive device under the action of external force. The other is the body resistance type, which the output electrical signal change depends on the body resistance change of the conductive material in the device under the external force. Flexible piezoresistive sensors have great advantages in terms of production and application. One is that the available materials and device structures are relatively rich. The other is that the data collection is difficult but flexible, which can collect current, resistance, and voltage as the output electrical signals.

### 2.2. Key Parameters of Pressure Sensors

In order to compare the performance of the sensor, researchers have stipulated a series of performance parameters as a standard to measure the performance of the sensor. These performance parameters include sensitivity, detection limit, range, linearity, response time, stability, etc.

#### 2.2.1. Sensitivity

Sensitivity (often represented by *S*) is one of the parameter standards for judging the sensing performance of flexible sensors, which can be expressed by Equation (2) [32]:

(2) $$S=\frac{\delta \left(\Delta E/E_{0}\right)}{\delta P}$$

where *E* is used to represent the electrical signal output by the flexible pressure sensor, *E*_0_ represents the initial electrical signal output, and *P* represents the pressure of the device. By calculating the ratio of the increase in the rate of change of the output electrical signal to the increase in the input pressure, the sensitivity dimension can be unified to kPa^−1^, thereby avoiding the difficult comparison between pressure sensors with different sensing principles due to different units. The sensitivity of a flexible piezoresistive sensor can be governed by Equation (3) [32,39]:

(3) $$S=\frac{\delta \left(\Delta I/I_{0}\right)}{\delta P}$$

where *I* is the current, Δ*I* is the change in current, and *I*_0_ is the initial current, or Equation (4) [32]:

(4) $$S=\frac{\delta \left(\Delta R/R_{0}\right)}{\delta P}$$

where *R* is the resistance, *R*_0_ is the initial resistance, and the sensitivity formula of the flexible capacitive pressure sensor is Equation (5) [32,40]:

(5) $$S=\frac{\delta \left(\Delta C/C_{0}\right)}{\delta P}$$

where *C* is the capacitance and *C*_0_ is the initial capacitance.

#### 2.2.2. Limit of Detection

The detection limit refers to the smallest pressure value that the device can measure [32]. The small detection limit can reflect the high sensitivity of the flexible pressure sensor to the applied external pressure, which is of great significance to the sensor in the field of robots, health detection and other areas that require small stress detection. The range of pressure from the smallest stress that the device can detect to the largest stress is the largest range that the sensor can detect. When other performance parameters are the same, a flexible sensor with large detecting range can be used in a richer field and applied to more scenarios.

#### 2.2.3. Linearity

Linearity (nonlinear error) is the degree to which the sensor sensitivity curve deviates from a straight line [32], usually expressed as $r_{L}\%$, and the linearity is decided by the formula below:

(6) $$r_{L}\%=\frac{\left|\Delta Y_{max}\right|}{Y}*100\%$$

where $\left|\Delta Y_{max}\right|$ is the maximum deviation in the vertical direction of the curve and *Y* is the maximum output value. The degree of linearity reflects the stability of sensor sensitivity [41]. In the linear range, the value of sensor sensitivity is more reliable. Therefore, the high sensitivity response with wide linearity in a large range is of great significance to the practical application of flexible sensors [32].

#### 2.2.4. Response Time

The time required for the sensor to respond to the electrical signal is the response time after a certain force is applied, the time required for the response process is from the initial state of the electrical signal to 90% of the maximum output state of the electrical signal or 1−1/e, and the time required for the sensor to recover is from the maximum state of the electrical signal to the initial state [32]. The response time is affected by the device structure, material, and stable environmental humidity. For example, if the material used in flexible sensor is viscous, there will be obvious response lag during the process of applying and unloading pressure, and the device response time and recovery time have a big gap.

#### 2.2.5. Stability

The stability and reliability of the sensor device in the application process is usually expressed by device stability [32]. If the electrical signal output by the device remains basically stable during a long period of loading and unloading at the same pressure under a room temperature environment, it can basically indicate that the device has cycle stability.

### 2.3. Strategies of High-Performance Devices

In order to improve the sensing performance of flexible pressure sensors for further practical application, researchers have developed a variety of performance control methods, which are mainly divided into two types; one is material selection and optimization, and the other is the design of force-sensitive interface micro-nanostructure. The key materials, structures and applications of flexible force sensors are illustrated in Figure 1.

The materials of flexible pressure sensors are mainly divided into flexible base materials, active electrode materials, and dielectric materials. In order to ensure the flexibility of the flexible pressure sensor, the materials used are all inherently flexible or are made into low-dimensional materials to achieve the purpose of flexibility. The commonly used flexible substrate materials are PDMS [13,42,43], PI [38,44], PU [45], PET [39,46], and other polymer materials. This is due to the fact that these polymer materials have stable performance and long polymer chains making the material tough and strong while ensuring flexibility. Compared with the choice of flexible substrate materials, the selection of conductive active materials, on the one hand, adheres to the principle of ensuring the flexibility of the device, and on the other hand, it also considers the conductivity of the material itself and the resistance change after force. Therefore, the commonly used active materials include low-dimensional materials and some compressible materials, so that the active electrode materials available for flexible pressure sensors are very abundant, such as metal materials, ordinary carbon materials, some emerging two-dimensional materials, and conductive organic materials. Metal materials also include various metal nanomaterials (nano-silver [47,48] and nano-gold [38,49]), metal thin-film materials (thin-film copper [50] and thin-film magnesium [51]), metal oxides [13], liquid metals [52], etc. Ordinary carbon materials include carbon nanotubes [53,54], carbon black [55], bio-derived carbon materials [56,57], etc. New two-dimensional materials include not only graphenes [40,58,59,60] and redox graphene [61,62] that have attracted much attention but also transition metal two-dimensional materials that have emerged in recent years (transition metal carbonitride Mxenes [63,64], two-dimensional transition metal chalcogenides TMDs [65]; conductive organic materials include PPy [66,67], PEDOT:PSS [68,69], etc. Owing to the large conjugated π bonds of conductive polymers, the electrons on the π bond can move in a large range and, thus, bring the polymer a conductive effect. As for the dielectric material, it is insulating. For flexible pressure–capacitance sensors, the dielectric material is often with a large dielectric constant. A large dielectric constant can make the sensor have a greater change in capacitance after being subjected to external pressure. The dielectric includes air [48], PDMS [40,70], PVA [71], PMMA [72], PVDF [73], etc. There are also high-permittivity materials such as P(VDF-TrFE) [50] and ZnO [40] added to the dielectric layer as the dielectric. Though there are many types of existing sensor materials, it is still necessary to develop new materials in spite of a huge amount of manpower and financial resources.

In addition to optimizing the materials of the flexible pressure sensor, the researchers also adjusted the performance of the flexible sensor by introducing microstructures into the sensor’s force-sensitive interface [59,61,68]. It is believed that the introduction of the interface microstructure changes the rate of change of the output electrical signal of the pressure sensor while improving the flexibility of the material itself. For flexible piezoresistive sensors, microstructures are often introduced on a flexible substrate. When the active electrode materials are attached to the flexible substrate, it also has microstructures, which not only increases the area occupied by the conductive active material but also reduces the initial current of the device due to the existence of the microstructures so that the flexible piezoresistive sensor has a large resistance change rate after being stressed. The introduction of microstructures on flexible substrates is also applicable to flexible pressure–capacitive sensors, but the principle of action is different from that of flexible piezoresistive sensors. The introduction of microstructures can increase the distance between the electrodes, so that the pressure sensor has a larger space for distance change under pressure and, at the same time, improves the dielectric constant of the sensor to achieve the purpose of changing the capacitance of the device. Some research groups have also introduced microstructures into the dielectric layer, and the principle of action is similar to the improvement on the substrate [50,70]. The microstructures often introduced in flexible pressure sensors can be divided into regular microstructures and random microstructures. Regular microstructures include pyramids [74,75], domes [76,77,78], rods [79,80], etc. These structures can be obtained by photolithography or template methods, but some specific equipment such as binary exposure machines are required. The required production cost is usually relatively high, and the graphics need to be designed in advance, which is relatively cumbersome. Random microstructure refers to the uneven size distribution of microstructures introduced into the flexible pressure sensor. This microstructure can be copied from the surface of other materials or obtained through simple operations, and the cost of production is relatively low. Common random microstructures can be obtained directly or indirectly from fabrics [81], plants [82,83], and sandpaper [84,85].

## 3. Regular Microstructures for Force-Sensitive Interface

### 3.1. Periodic Micro-Nanostructures

The performance of flexible pressure sensor is often adjusted by introducing periodic micro-nanostructures to the force-sensitive interface of the flexible pressure sensor. The commonly used periodic regular micro-nanostructures include pyramids [40,49,68,70,95], domes [76,96], rods [59,89,97,98], etc. These microstructures can be prepared by lithography, soft lithography, laser processing, etc.

#### 3.1.1. Lithography and Soft Lithography

Lithography is a common method in micro-nano-processing technology. Chen et al. fabricated micropillar arrays on Si wafers by photolithography, magnetron-sputtered a 100-nm Au conductive layer on the Si wafers, and integrated it with a conductive PPy polymer film. The sensitivity of the obtained flexible piezoresistive sensor is as high as 17 kPa^−1^, and the sensitivity of the device can be adjusted by changing the size of the micro-columnar structure [97]. 

**Figure 2:**
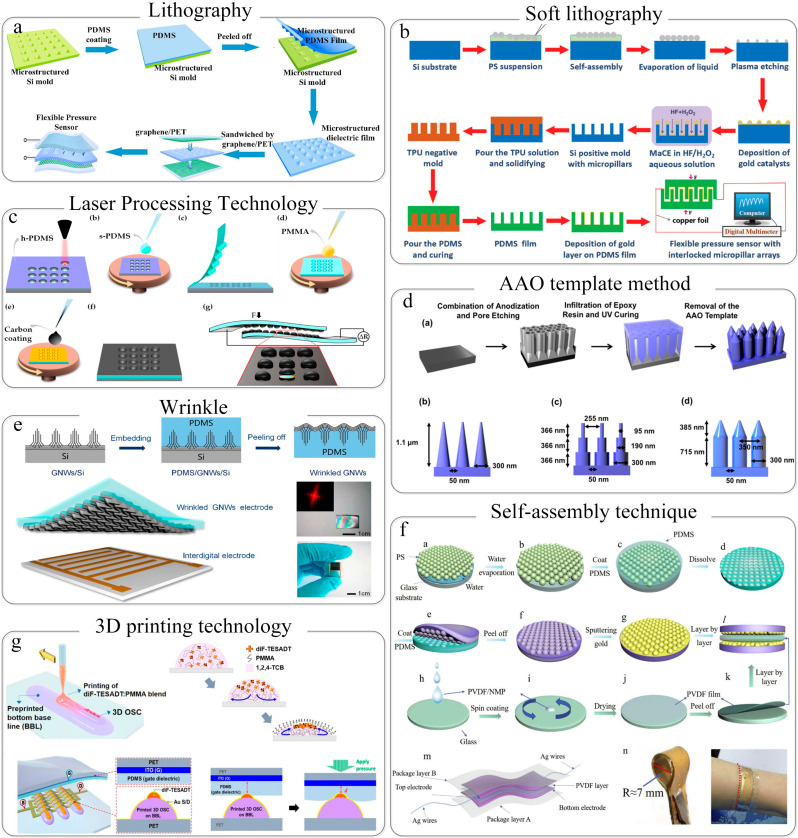
Methods for periodic/quasi-periodic force-sensitive structure to regulate the performance of flexible pressure sensor. (**a**) Lithography. Reprinted with permission from reference [70]. Copyright © 2018 Elsevier Ltd. (**b**) Soft lithography. Reprinted with permission from reference [80]. Copyright © 2019 WILEY-VCH Verlag GmbH & Co. KGaA, Weinheim. (**c**) Laser processing technology. Reprinted with permission from reference [96]. (**d**) AAO template method. Reprinted with permission from reference [106]. Copyright © 2018, American Chemical Society. (**e**) Wrinkle. Reprinted with permission from reference [39]. Copyright © 2021, American Chemical Society. (**f**) Self-assembly technology. Reprinted with permission from reference [73]. Copyright © 2020 Elsevier Ltd. (**g**) 3D printing technology. Reprinted with permission from reference [108]. Copyright © 2017, American Chemical Society.

Photolithography technology can prepare uniform and periodic microstructures, but the required equipment is expensive and the preparation steps are relatively cumbersome. If the microstructures are directly prepared on the substrate by the photolithography technology, it is difficult to prepare the microstructures on a large area, and the use efficiency of substrates containing microstructures is also low. Therefore, in addition to preparing periodic microstructures directly on the substrate by photolithography, many research groups also first use photolithography to prepare microstructures on harder materials, such as Si wafers, as microstructure templates, and then, the elastic material is used to replicate the microstructure in the template, which not only ensures the flexibility of the sensing device by making a periodic microstructure but also allows the template to be used repeatedly, greatly improving the use efficiency of the same template. For example, Wei et al. prepared a micro-pyramid structure on a Si wafer, replicated the microstructure through PDMS elastomer, and used it as the dielectric layer of a flexible capacitive pressure sensor (Figure 2a) [70]. The research group also used photolithography to make a Cu template on Cu foil with a micropillar structure and then grew graphene on the Cu template by a plasma enhanced chemical vapor deposition (PECVD) method to replicate the microstructure on the PDMS elastomer. At the same time, graphene and microstructure can be combined to achieve a three-dimensional conformal state of the graphene microstructure [89]. By integrating these two conformal films and PET/UVA dielectric into a flexible pressure sensor, the sensitivity of the device reaches 7.68 kPa^−1^. Recently, this group also announced a work on the preparation of high-performance flexible capacitive pressure sensors through photolithography templates [40]. The difference from the previous work [70,89] is that this work is based on the preparation of the microstructure/conformal thin film of conductive active material from the photolithography template, and the dielectric layer is also integrated by spin coating and sputtering. On the surface of the microstructure, a conformal film of the substrate microstructure/conductive active material/dielectric is formed. The sensing performance of the flexible pressure–capacitance sensor obtained through this film is greatly improved (sensitivity: 22.3 kPa^−1^).

Wong et al. prepared a pre-template with micro-columnar structure through technology-assisted chemical etching (MaCE) and then copied the microstructure in the pre-template as a template through TPU elastomer and, finally, used PDMS to remove the microstructure of the template. The rod-shaped structure is transferred, and gold is sputtered on the surface of the PDMS with microstructures. The two pieces of PDMS film packaging can be integrated into a flexible pressure sensor with piezoresistive effect, as shown in Figure 2b [80]. The method of transferring the microstructure from the elastic template to the flexible sensor substrate like this is collectively referred to as soft lithography. The advantage is that the process of transferring the microstructure to the substrate does not involve the exposure and etching in photolithography. Besides, the cost is low, and the production is relatively convenient. Similarly, the pyramid microstructure can also be prepared by soft lithography [99]. This work obtained through theoretical simulations that the pyramid structure is the optimal shape and also explored the influence of the microstructure spacing on the sensor sensitivity (too large or too small a distance is not the best choice to increase the sensor’s sensitivity). Nanoimprint technology is included in the soft lithography technology. Owing to nanoimprint technology using electron beams to prepare microstructures, the precision of the prepared microstructure template is higher than that of the template prepared by photolithography, and it can be prepared in a large area [98]. Li et al. used a PDMS mold with a micro-rod structure to imprint P (VDF-TrFE) film on a micro-nanostructure, thereby improving the dynamic sensing performance of the flexible pressure sensor [100]. Compared with the three-dimensional structure, the two-dimensional microstructure produced by imprinting technology is smaller in size and has higher stability [101]. Sun et al. reported a method of assembling gold nanoparticles into tightly arranged and densely stacked micro/nanowires by imprinting, which can be used for high-performance flexible pressure sensors [101]. Due to the fact that the nanometal particles are integrated into very thin nanowires, the metal particles are very dense and provide a lot of contact points, so the flexible sensor prepared therefrom can work under low pressure. 

#### 3.1.2. Laser-Induced Structures

In addition to lithography technology and soft lithography technology, laser processing technology is also a technology used to manufacture flexible pressure sensors; that is, processing materials by using laser beams to interact with substances. Duchaine et al. used a CO_2_ laser to prepare periodic microstructures on acrylic materials as a microstructure template at a speed of 600 dpi and poured a silicone resin and BaTiO_3_ composite onto the acrylic template for curing to obtain the electric layer with double-layer microstructures of the flexible pressure sensor [102]. The microstructure constructed by the CO_2_ laser has double layers, which is also a very prominent advantage of laser processing technology-without expensive means such as photolithography. What is more, the laser beam can be controlled to complete the pattern by need in a large range, which can give the flexibility sensor a larger performance variation range, and the silicone resin/BaTiO_3_ composite used enhances both the dielectric constant of the dielectric layer and the capacitive response of the flexible pressure–capacitance sensor. Similarly, Igreja et al. used laser processing technology to engrave micro-cone structures on acrylic materials [103] and carve a hemispherical structure on the hard PDMS (Figure 2c). [96] Xuan et al. laser microfabricated micro-cone structures on PDMS materials [104]. These are all required microstructures engraved on the materials by laser processing technology as the microstructure template of the sensor.

The lithography and soft lithography technology introduced above, including laser processing technology, are relatively complicated. Although the required periodic/quasi-periodic structure can be prepared, specific equipment is inevitably required, such as binary exposure machines, etching machines, etc. They are all very expensive, and the structure needs to be designed, which takes a lot of time to complete.

### 3.2. Quasi-Periodic Micro-Nanostructure

Since most of the commonly used methods for preparing periodic micro-nanostructures are time-consuming and the required equipment is expensive, the prepared templates need to be reused in order to reduce costs, and it is inevitable that there will be residual materials in the recycling process, which will affect subsequent use. For the sake of avoiding the above shortcomings, researchers have developed some alternative methods for the preparation of micro-nanostructures, such as AAO template method, folds, and self-assembly. The micro-nanostructures prepared by these methods are not completely periodic, so we usually refer to these micro-nanostructures as quasi-periodic micro-nanostructures.

#### 3.2.1. AAO Template Method

The anodic aluminum oxide (AAO) template method is a common method to introduce microstructures into flexible pressure sensors. Usually through the anodizing process; using sulfuric acid, oxalic acid, and phosphoric acid as electrolytes; and controlling different electrolysis voltage, time and frequency, AAO templates with different shapes and appearances (rod, cone, step, and pencil); spacing; and size can be prepared [37]. For example, Yang et al. applied a fixed voltage of 160 V on hard anodizing, controlled the anodization to dominate the vertical growth of pores at a rate of 0.5 to 0.6 nm s^−1^, and finally, precisely regulated the shape of the AAO template [105]. Using the same method, the research group also successfully prepared three nanostructures (cones, ladder-shaped, and pencil-shaped nanostructures) with a diameter of 300 nm and a height of 1.1 μm, as shown in Figure 2d. The pencil-shaped nanoparticle array in this microstructure showed the highest shear adhesion strength in all indentation depth tests through the finite element analysis, which is consistent with the experimental results [106]. This is ascribed to the different strengths of materials exhibited by different microstructure shapes. The pencil-shaped nanoparticle array has higher bending stiffness and tighter arrangement, so that the shear adhesion strength of the material increases with the increase of the indentation depth. In addition to exploring the relevant properties of the microstructure obtained from the AAO template, many research groups also applied it to the preparation of flexible pressure sensors. Li et al. used the AAO template method to prepare P (VDF-TrFE) into a dielectric layer with microrod-like structures on both sides and integrate it with copper foil to form a flexible capacitive pressure sensor [44]. The pressure sensor prepared by this method has high sensitivity (~0.35 kPa^−1^), wide working range (4 pa~25 kpa), short response time (~48 ms), and excellent durability. By simulating the tactile structure of a human finger, Liu et al. prepared P (VDF-TrFE) into a micro-vertebral structure and attached it to a Cu foil and then encapsulated the microstructure of the film “face-to-face”. Through this modification, the sensitivity of flexible pressure sensor has been greatly improved (sensitivity: 6.583 kPa^−1^) [50]. The preparation of flexible pressure sensors by AAO template does not require expensive mechanical equipment, so the overall production cost is low. The required templates can be prepared on a large scale by controlling the production parameters, which is easy to mass produce the sensor.

#### 3.2.2. Wrinkle Method

Templates are used in many methods of preparing microstructures, such as soft lithography and AAO template methods. These template methods require troublesome pre-design or post-processing. As a three-dimensional microstructure, wrinkles can be directly introduced into the flexible pressure sensor by simple means such as pre-stretching, in situ growth, and heat treatment. Hong and his partners pre-stretched the PDMS elastomer and treated the surface with UV and O_3_, then coated with silver nanowires and released it to obtain PDMS/AgNWs elastomer with wrinkles on the surface. Finally, the microstructure was replicated by PDMS solution and obtained an elastic substrate that not only has a wrinkled structures but also combines well with AgNWs [72]. The flexible pressure sensor obtained by this method shows good flexibility and stability. Moreover, the sensitivity of it is >3.8 kPa^−1^, and the response time is <150 ms. Lu et al. self-wrinkle-grown multi-scale nested PPy films in situ on PDMS substrates in a mixed acid solution [66]. The “face-to-face” packaging of this wrinkled PPy/PDMS film, due to the material stress relief surface characteristics and the self-adaptive ability of the surface wrinkle shape, makes the flexible pressure sensor a good performance (the sensitivity: 19.32 kPa^−1^, the fast response time: <30 ms, and the low detection limit: 1 Pa). In addition, Yang et al. used a simpler thermally induced wrinkle method (Figure 2e), only changing the heating temperature required for the transfer of GNWs to PDMS elastomers to prepare GNWs/PDMS films. Then, the GNWs film and the interdigital electrode are integrated into a flexible pressure sensor with good performance (a high sensitivity: 59.0 kPa^−1^) [39]. The size of wrinkles prepared by stretching, in situ growth, or heat treatment can be changed by controlling the corresponding parameters, but the structure mode obtained is relatively simple.

#### 3.2.3. Self-Assembly Method

Self-assembly technology is a low-cost micro-nano-processing technology that can prepare microstructures in a large area. Wong et al. used monodisperse polystyrene (PS) microspheres to self-assemble into a single-layer and ordered sacrificial template. By pouring PDMS solution onto the template and removing the PS microspheres after solidification, a flexible substrate with microsphere structure can be obtained [107]. The size of the microstructure in this flexible sensor can be changed by adjusting the size of the PS microspheres. The resulting micro-structured thin film was coated with a gold layer by magnetron sputtering and then aligned the packaging. The sensitivity of the as-prepared flexible piezoresistive sensor can be as high as 15 kPa^−1^. Moreover, this sensor has been successfully applied to measure human neck pulse, showing a wide range of application prospects in human health monitoring. Similarly, the research group used the same PS microspheres to self-assemble into a single-layer sacrificial template. After microstructure replication and transfer, a flexible PDMS substrate with microstructures was obtained. The surface of the PDMS substrate microstructure was sprayed with gold, and the thin PVDF film acted as a dielectric layer (Figure 2f), which integrated a flexible pressure sensor [73]. This sensor with excellent flexibility and sensing performance (sensitivity: 30.2 kPa^−1^) can monitor various human motion signals and physiological signals, which has great application potential in the wearable field. Self-assembly technology has obvious advantages (low cost and easy large-area preparation of microstructures), but its disadvantages are also obvious. Successfully prepared microstructure templates usually can only be used once, and the template utilization efficiency is low.

### 3.3. 3D Printing Structure 

As an emerging micro-nano-processing technology, 3D printing has developed rapidly in recent years and been applied in the manufacture of flexible pressure sensors. 3D printing technology not only prints microstructure templates but also directly prints materials. For example, Guo et al. used 3D printing technology to print an acrylonitrile–butadiene–styrene (ABS) template with periodic microgrooves and then used the template to replicate the PDMS dielectric layer with periodic microgrooves [47]. Wang et al. used 3D printing technology to print a variety of microstructure templates, including microspheres, pyramids, and microgrooves [90]. Lim et al. directly 3D printed the material on the device to realize the 3D of organic materials (Figure 2g) [108]. Three-dimensional printing can quickly print out the required templates through 3D printing equipment, but the resolution of the microstructures printed by existing printers is low [109], which means that it is not suitable for the miniaturized integrated manufacturing of flexible sensor.

## 4. Irregular Microstructures for Force-Sensitive Interface 

### 4.1. Bulk Filling Structure 

**Figure 3:**
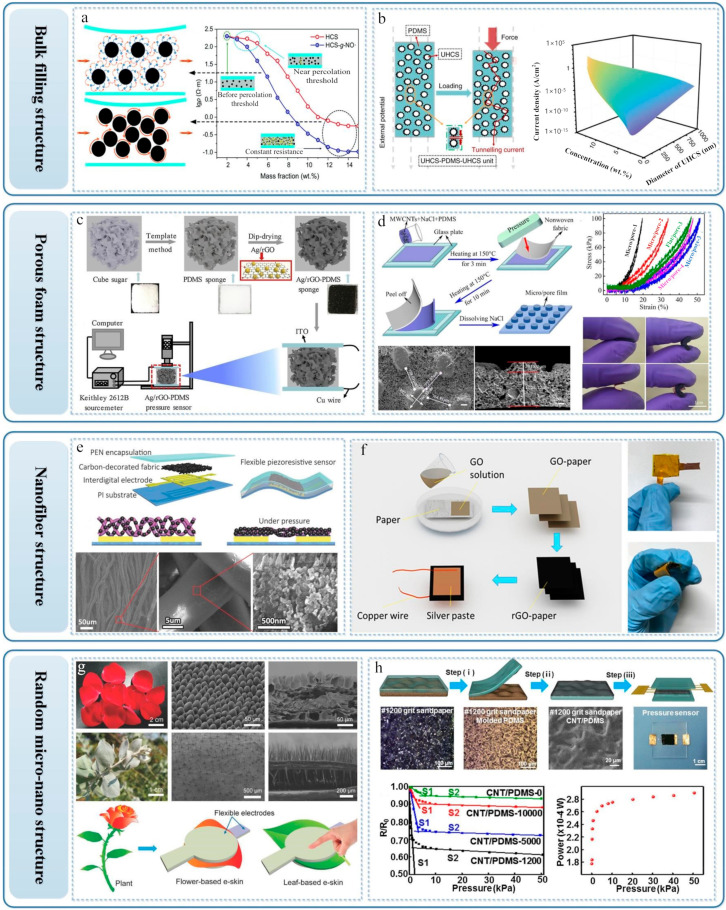
Methods for disordered force-sensitive structure to regulate the performance of flexible pressure sensor. Bulk filling: (**a**) hollow carbon nanospheres as bulk filling materials. Reprinted with permission from reference [86] (Copyright 2020, RSC Pub). (**b**) Sea urchin-like carbon balls as bulk filling materials. Reprinted with permission from reference [111] (Copyright © 2020, The Author(s)). Porous foam structure: (**c**) sugar cubes as foam templates. Reprinted with permission from reference [117] (Copyright © 2019 Elsevier B.V. All rights reserved.). (**d**) NaCl as a foam template. Reprinted with permission from reference [116] (Copyright © 2021, American Chemical Society). Nanofiber structure: (**e**) carbon-decorated fabric as sensor active material. Reprinted with permission from reference [119] (Copyright © 2015 WILEY-VCH Verlag GmbH & Co., KGaA, Weinheim). (**f**) rGO-modified paper as active material. Reprinted with permission from reference [77] (Copyright © 2017, American Chemical Society). Random micro-nanostructure: (**g**) the leaves/petals of plants act directly as the dielectric layer. Reprinted with permission from reference [48] (Copyright © 2018 WILEY-VCH Verlag GmbH & Co., KGaA, Weinheim). (**h**) Sandpaper as a template for random microstructures. Reproduced with permission from reference [123] (Copyright © 2018 Elsevier B.V. All rights reserved).

The researchers filled the conductive material into the elastic material, and the deformation of the elastic material after pressure causes the inner filling material contact. By this way, the conductive path or generate the quantum tunneling effect increases, thus leading the current increases. Since the disordered bulk filling material can produce the force-sensitive effect of the contact interface after being pressed, here, we summarize it as the regulation of the performance of the flexible pressure sensor by the disordered force-sensitive interface. Wang et al. explored the piezoresistive properties of the composite obtained by combining carbon nanotubes and silicone rubber [110]. The study found that the monotonicity of the composite resistivity is related to the content of carbon nanotubes and the range of applied pressure. It has been studied that the carbon nanotubes concentration of 18% is a dividing line. When the concentration is lower than 18%, the resistance increases monotonously with the increase of pressure. When the concentration is higher than 18%, the resistance of the composite will first decrease and then increase with the increase of pressure, which provides some ideas for the preparation of flexible piezoresistive sensors by bulk filling method. In order to improve the sensing performance of the bulk-filled structure sensor, Cai et al. used hollow carbon spheres as conductive fillers to obtain composite materials with piezoresistive sensing, and modified the hollow carbon spheres with nitro radicals to explore the piezoresistive performance of the composite (Figure 3a) [86]. Afterwards, Wu et al. changed the filling material and prepared the filled conductive material into PDMS by the shape of sea urchin-like carbon balls. The structure of such carbon balls itself has a convex tip, which can generate tunneling current between the carbon balls when under pressure (Figure 3b). Moreover, due to the low filling concentration of the material, the carbon ball/PDMS composite exhibits high transparency characteristics [111]. In addition to directly filling conductive particles into the composite to form bulk filling materials with piezoresistive effect, some research groups embedded conductive network structure into the composite to control the sensor performance [112,113]. For example, Cheng et al. fabricated a three-dimensional graphene network structure using the network structure of Ni foam and then immersed the graphene network in the elastic solution of PDMS to generate a soft and stable flexible sensor [112]. The preparation of flexible sensors by bulk filling is low-cost and easy to operate, but its shortcomings are also very obvious. One is that it is difficult to mix conductive materials and flexible polymer materials, and the other is that the prepared sensors are bulky and difficult to array.

### 4.2. Porous Foam Structure

The flexible pressure sensor prepared by the porous foam structure deforms in the foam void during the compression process, and the conductive material around the void contacts to increase the conductive path of the device, thereby changing the output electrical signal of the sensor. There are three common methods for preparing porous foam: freeze-drying method [114,115], salt or sugar template method [116,117], and sponge surface-coating method.

The freeze-drying method allows the material to be solidified at a low temperature while maintaining the porous structure of the material. Li et al. prepared graphene-based porous elastomers by freeze-drying, which has the characteristics of fast dynamic piezoresistive response [114]. The graphene-based elastomer prepared by freeze-drying is in the shape of a sheet. During the compression process, the graphene sheets in the foam contact to reduce the overall resistance of the material. Sun et al. used the one-pot method and freeze-drying method to prepare copper nanowire aerogels and, for the first time, proposed the “foam-controlled assembly” mechanism, which can adjust the pore structure and density of the aerogel [115].

The salt/sugar template method is also a common and simple method to make porous foam. By mixing salt or sugar into the material, the salt/sugar is dissolved after the material is solidified and shaped to obtain the porous foam. Peng et al. used sugar cubes as a template, soaked the sugar cubes in PDMS solution and solidified them to prepare a porous foam, and then coated the Ag/rGO composite conductive material on the surface of the porous foam by soaking to obtain a conductive foam (Figure 3c) [117]. Zhu et al. mixed NaCl with PDMS and multi-walled carbon nanotubes (MWCNTs) uniformly and then used nonwoven fiber cloth to make a three-dimensional micro-columnar structure and finally dissolved NaCl to obtain a porous PDMS/MWCNT film with a surface microstructure (Figure 3d) [116].

The sponge surface coating method refers to the use of a nonconductive sponge as a template, and the sponge surface is coated with a conductive material by soaking [118]. Yu et al. used PU sponge as a template, soaked the surface of the PU sponge with graphene oxide (GO), then reduced the GO to rGO in the atmosphere of the HI hot solution, and finally created a dense fracture microstructure in the atmosphere of heat and pressure to improve the pressure sensitivity of the foam [45]. The high-density fractured microstructure PU foam (RGO-PUS-HT-P) prepared in the experiment is attached with copper electrodes on both sides to make a flexible piezoresistive sensor. The sensitivity of the sensor is 0.26 kPa^−1^ within 0–2 kPa.

Although the method for preparing a flexible pressure sensor from porous foam is relatively simple, it is necessary to consider the volume and flexibility of the completed device. In the case of limiting the volume of the porous foam used, the range of resistance of the device is also restricted, which means the sensitivity of the flexible pressure sensor is not high accordingly.

### 4.3. Nanofiber Structure

The preparation of some flexible pressure sensor improves the sensitivity of the pressure sensor by using the nanofiber structure of fiber, paper, or fabric. For example, Zhao et al. used carbon black to decorate fabrics and made a flexible pressure sensor that deforms the fiber under the action of force. This sensor can be used to measure blood pressure (Figure 3e) [119]. Zhang et al.’s fabric was immersed in Mxenes solution to prepare Mxenes fabric and packaged with Mo electrodes. The resulting flexible pressure sensor has a sensitivity of 12.095 kPa^−1^, which has the potential to be applied to wearable devices and human–computer interaction [120]. In addition, Ren et al. used paper as a substrate, dripped GO on the surface of the paper, reduced it to obtain rGO-paper conductive material, and then packaged it to obtain a simple flexible piezoresistive sensor (Figure 3f) [77]. Liao et al. carbonized the layered fluffy cotton and soaked it in PDMS to solidify to prepare a carbonized cotton-PDM composite and obtained a flexible pressure sensor with excellent flexibility [91]. The direct use of fiber materials as the source of the flexible sensor micro-nanostructure greatly reduces the cost required for preparation. What is more, it can be prepared into everyday clothing and directly applied to human wearable devices.

### 4.4. Random Micro-Nanostructure 

Human beings are inspired by the structures in organisms and have obtained many random micro-nanostructures that can be applied to flexible pressure sensors. We call these structures a unified biomimetic structure. Using organisms as templates can introduce microstructures into flexible pressure sensors, and existing studies include plant leaves [48,82,83,121], insect wings [122], animal skins [36,69], etc. Among the above organisms, plant leaves are commonly used in research. Zhang et al. used common indoor plant leaves as templates, replicated the microstructure of the leaves through PDMS, and prepared a flexible pressure sensor with a sensitivity of 19.8 kPa^−1^ by arranging carbon nanotubes/graphene (ACNT/G) as the active material [121]. Gao et al. used Ginkgo biloba with a denser structure as the template, replicated the microstructure through PDMS, and encapsulated the Mxene microstructure film and interdigital electrodes with Mxene as the active material. The resulting flexible pressure sensor has a sensitivity of 403.46 kPa^−1^ [64]. The reason for the higher sensitivity of the sensor of Gao’s research group may lie in: (1) the choice of bionic microstructure templates, the denser and richer microstructure of Ginkgo biloba itself; (2) the choice of active materials, Mxene material is a two-dimensional multilayer structure, so it may be more sensitive to force; and (3) the counter electrode used is an interdigital electrode instead of the same electrodes on both sides. When interdigital electrodes are used, the electrodes on both sides of the sensor remain hard on one side and soft on the other side. When an external force is applied to the PDMS, the microstructure changes greatly, and the corresponding device resistance changes more. In addition to using plants as templates to replicate the release structure, there is also the direct use of plants as the dielectric layer of the flexible capacitive pressure sensor. Guo et al. used plant leaves and petals directly as the dielectric layer. Due to the presence of 3D microstructures on the leaves and petals, they will deform after being pressed. Therefore, the obtained flexible pressure-capacity sensor has good sensing characteristics with a sensitivity of 1.54 kPa^−1^ (Figure 3g) [48].

In addition to the bionic microstructures provided by natural organisms, artificial sandpaper is also often used as a template for microstructures. Xuan et al. used sandpaper as a template, PDMS as a flexible substrate to replicate the random microstructure of sandpaper, and chose CNTs as the active material. The prepared flexible piezoresistive sensor has 20 times higher sensitivity than a sensor without microstructure (Figure 3h) [123]. Subsequently, by adjusting the number of sandpaper meshes, the research group studied the sensing performance of the corresponding flexible piezoresistive sensors under different mesh numbers. The study found that the sensitivity of the sensor was the highest when the number of sandpapers was 1200 meshes, which was 0.20917 kPa^−1^. This is because when the mesh is too large, the microstructure is too small, which is not sensitive to pressure. When the mesh is too small, the resistance of the device changes little during the pressure process. Zeng et al. made a flexible piezoresistive sensor with excellent performance by replicating the microstructure of sandpaper, using rGO as the active material and the interdigital electrode as the counter electrode [62]. The sensitivity of this sensor is as high as 1051 kPa^−1^, and the measuring range is 0.01–400 kPa. Due to the random multilevel microstructure of the sandpaper, the sensor still maintains a high sensitivity at a large range.

## 5. Sensor Designs with Force-Sensitive Interface Engineering

### 5.1. Single-Sided Force-Sensitive Structure

**Figure 4:**
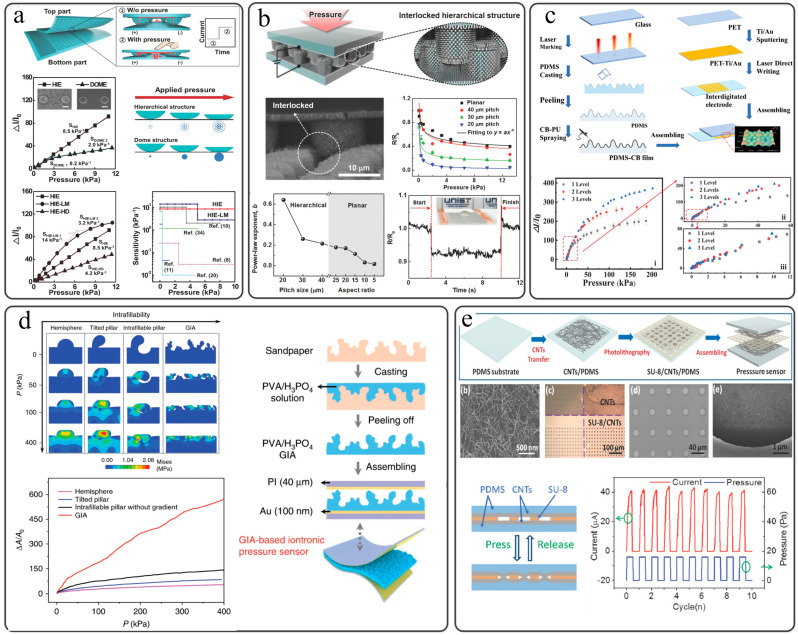
Method for adjusting the performance of flexible pressure sensor through device structure design. (**a**) Single-sided force-sensitive structure. Reprinted with permission from reference [61]. Copyright © 2016 WILEY-VCH Verlag GmbH & Co., KGaA, Weinheim. (**b**) Interlocking force-sensitive interface. Reprinted with permission from reference [13]. Copyright © 2015 WILEY-VCH Verlag GmbH & Co., KGaA, Weinheim. Micro-nano-multilevel structure: (**c**) Micro-nano-multilevel structure obtained by laser marking method. Reprinted with permission from reference [55] (Copyright © 2021 Wiley-VCH GmbH), (**d**) The micro-nano-multilevel structure obtained by sandpaper is used for the dielectric layer. Reproduced with permission from reference [38]. Copyright © 2020, The Author(s). (**e**) Spacer setting of force–electric coupling interface. Reproduced with permission from reference [54]. Copyright © 2017 WILEY-VCH Verlag GmbH & Co., KGaA, Weinheim.

The single-sided force-sensitive structure is the most common device structure among flexible sensor devices. Choong et al. introduced a micro-pyramid array into one side interface of the flexible piezoresistive sensor device by photolithography, and the other side of the interface used a counter electrode with no microstructure [68]. Since the device structure of the single-sided force-sensitive structure is relatively simple, the actual working principle of the sensor is relatively simple, and there is no need to consider the influence of the contact mode between the microstructures on the performance of the sensor [15]. Cho et al. used the Cu biological hierarchical structure template obtained by photolithography to fabricate the sensor force-sensitive surface, using rGO as the conductive active material of the force-sensitive surface and using the interdigital electrode as the counter electrode of the force-sensitive surface. The resulting best sensitivity of flexible sensor is 6.8 kPa^−1^ (Figure 4a) [61]. Yang et al. also fabricated the force-sensitive surface of the micro-pyramid structure by photolithography and fabricated a PDMS dielectric layer on the surface of the force-sensitive surface by spin coating and sputtered on high-permittivity ZnO to improve the dielectric layer. The dielectric constant makes the dielectric layer and the conductive layer form a three-dimensional conformal structure. By using the PET plane with AgNWs as the counter-electrode, a high-performance (22.3 kPa^−1^, 22 kPa) flexible pressure–capacitance sensor is prepared [40]. Gao et al. directly used sandpaper as a template to make a flexible substrate with random microstructures, sprayed Mxene as a conductive material on the surface of the flexible substrate, and then used a flat interdigital electrode as a counter electrode to encapsulate a flexible sensor [63]. The sensitivity of this sensor is as high as 151.4 kPa^−1^, the low detection limit is 4.4 Pa, and it has good stability (>10,000 cycles). Although some flexible pressure sensors with single-sided force-sensitive interface also have good sensing performance, the structure of single-sided force-sensitive interface is relatively simple, which makes the measuring range of the sensor small.

### 5.2. Interlocked Force-Sensitive Interface

Humans get a lot of inspiration from nature for the design of flexible sensors. An important reason for the high sensing efficiency of human fingers is the interlocking structure in the finger skin [78]. Therefore, the researchers introduced the interlocking structure into the structure of the flexible force-sensitive sensor device to improve the sensor’s sensing performance. There are many types of microstructures used to form interlocking structures, including nano-hair [124], rods [13,15,125], domes [76,126], pyramids [50], nano-spring [127], etc. Suh et al. prepared a nano-hair array by a template method. The surface of the microstructure film was coated with a Pt coating to realize the device conduction. The microstructures of the two films were relatively encapsulated, and the nano-hair array formed an interlocking force-sensitive interface [124]. On account of density and softy of nano-hair structure, it is very easy to undergo various deformations under the action of force. Therefore, this flexible piezoresistive sensor can detect pressure, tensile force and torsion force. Ko et al. designed an electronic skin that can detect static and dynamic pressure [13]. The micro-columnar structure in this electronic skin is prepared by photolithography technology. On the microstructure, vertically aligned zinc oxide nanowires are grown by in situ growth. The micro-nanostructure and the ZnO nanowire array form a biologically inspired interlocking-layered structure (Figure 4b). Liu et al. prepared a quasi-periodic micro-tapered dielectric layer by the AAO template method and aligned and packaged the two dielectric layer microstructures to obtain a flexible piezocapacitance sensor with interlocked dielectric layers. The sensitivity of the sensing device is 6.583 kPa^−1^ [50]. The inspiration of the interlocking structure comes from human and nature, and how to introduce the interlocking structure into the flexible pressure sensor also brings researchers more ideas to develop new sensor structures.

### 5.3. Micro-Nano-Hierarchical Structures

The flexible piezoresistive sensor with a single microstructure is easy to reach current saturation under the action of external pressure, while the micro-nano-multilevel structure has a variety of microstructures. The small-sized microstructure under high pressure can play a vital role in current compensation and make the pressure sensor have a large range, which also has high sensitivity in a large range. The source of the micro-nano-multilevel structure can be sandpaper, wood, etc. For example, Zeng et al. used sandpaper to obtain a PDMS flexible substrate with a random micro-nano-multilevel structure and used rGO as a conductive active material to prepare a high-sensitivity, large-range flexible pressure sensor (2.5–1051 kPa^−1^, 0.01–400 kPa) [62]. Wang et al. prepared a wood-based high-performance flexible piezoresistive sensor by chemical treatment and modified flexible wood (FW/rGO) with rGO [128]. The advantage of this kind of flexible pressure sensor is that it is very environmentally friendly without the use of nondegradable high-molecular synthetic polymers. The linear range of the sensor is as high as 60 kPa, and the sensitivity is 1.85 kPa^−1^. Due to the fact that the micro-nano-multilevel structure obtained from the outside world cannot control the distribution of the structure, some research groups process the required micro-nano-multilevel structure by artificial means. Zhou et al. prepared flexible piezoresistive sensors with multilevel structures through low-cost laser marking technology, template technology, and spraying technology [55]. As shown in Figure 4c, the desired ordered micro-nano-multilevel structure can be prepared by laser marking technology, and the sensing performance of the flexible sensor can be adjusted.

For flexible pressure–capacitance sensors, the main advantage of the micro-nano-multilevel structure is that the large-size micro-nanostructure can improve the sensitivity of the sensor, and the small size can reduce the hysteresis caused by interface adhesion [95]. Pan et al. used photolithography and anisotropic wet etching to prepare micro-nanostructures of different sizes on Si wafers and replicated PDMS flexible substrates with multilevel microstructures using Si wafers as templates [95]. The flexible sensor prepared by the flexible substrate of this micro-nano-multilevel structure has a very low hysteresis rate (4.42%) and a relatively high sensitivity (3.73 kPa^−1^). For the ionic flexible pressure–capacitance sensor, since the contact surface of the active dielectric layer and the electrode forms an electric double layer after pressure is applied to improve the capacitance response of the sensor, the micro-nano-multilevel structure can significantly improve the compressibility of the structure and pressure response range [38]. Guo et al. used sandpaper as a template (Figure 4d) and PVA/H_3_PO_4_ as a dielectric layer to prepare a flexible capacitive sensor with extremely high sensitivity (Smin > 220 kPa^−1^) and a wide range (0.08 Pa–360 kPa) [38]. The convex structure of the dielectric layer provides the possibility of high sensitivity of the flexible pressure sensor. In addition, the fillable structure of the dielectric layer enables the device to maintain stability during the deformation process and increases the range of force that the sensor can detect. In general, the micro-nano-hierarchical structures contained in the force-sensitive interface can greatly improve the sensitivity of the sensor and modify the overall sensing performance of the device, which may have important guiding significance for the future development of flexible pressure sensors.

### 5.4. Spacer Design of Force-Sensitive Interface

The sensitivity of the flexible piezoresistive sensor is calculated by the Formula (3).

It can be seen that the sensitivity is related to the initial current and current change of the sensor. Without changing the conductive active material, in addition to introducing a microstructure to adjust the device current, the researchers also proposed a spacer layer structure with a mechanical–electric coupling interface. Gui et al. used the carbon nanotube conductive network as the conductive material and the insulating network formed by the SU-8 photoresist as the dielectric layer to prepare a scalable and integrated pressure sensor based on contact resistance [54]. This device gets rid of the traditional high-sensitivity sensor preparation method, which can be easily integrated with silicon-based microelectromechanical systems (MEMS). It has high sensitivity (95.5 kPa^−1^), low detection limit (16 Pa), and its power consumption is 0 when there is no pressure (Figure 4e). In addition, the output current of the piezoresistive sensor can be controlled by adjusting the aperture and thickness of the photoresist insulating layer, thereby controlling the sensitivity of the sensor. The preparation of the insulating layer by photolithography requires expensive equipment such as binary exposure machines and etching machines, so Gao et al. ruled out this method and spun PVA fibers directly on the interdigital electrodes through electrospinning. The synergistic adjustment effect of PVA fiber, interdigital electrode, and wrinkled graphene film forms a force–electric coupling interface, thereby fabricating a high-sensitivity flexible pressure sensor [71]. It is worth noting that the thickness of the dielectric layer needs to be considered in the process of preparing a highly sensitive flexible piezoresistive sensor by adding a spacer layer, and a too-thick spacer layer will make the device nonconductive. Gui et al. used this principle to choose thick nylon and graphene to prepare a capacitive pressure sensor that responds quickly to dynamic interactions [129]. By controlling the spacer layer, a high-sensitivity flexible piezoresistive sensor can be obtained. Kuo et al. used SEBS nanofibers woven into a fiber-interlocked fabric as a dielectric layer and integrated it with an electrode layer made of fabric/AgNPs composite into a stretchable full-fiber flexible piezoresistive sensor [130]. This sensor has a high sensitivity of 71.07 kPa^−1^, and because of its full-fiber structure, it has great potential for application in the field of wearable devices. Recently, Zhao et al. woven a grid-like polyurethane fiber-based spacer layer through near-field electrospinning and explored the influence of the period and thickness of the spacer layer on the sensitivity of the sensor [53]. Under optimal conditions, the sensitivity of this flexible pressure sensor is 1.91 kPa^−1^, and the sensor’s sensing range can reach 230 kPa. The spacer design of the force-sensitive interface enables improved flexible pressure sensing performance at low cost without changing the device material and micro/nanostructure.

### 5.5. Brief Summary

There are many types of force-sensitive interface microstructures of flexible pressure sensors. According to whether the microstructure of the device interface is regular or not, the microstructure of the force-sensitive interface can be divided into regular microstructures and irregular microstructures, which can be introduced into the sensor by various methods. Table 1 summarizes some representative flexible pressure sensors fabricated by various methods and their performance indicators. As can be seen in Table 1, many research groups focus on exploring the sensitivity of flexible pressure sensors with interface microstructures but often ignore some important performance parameters such as limit of detection (LOD) and response time. Additionally, the overall performance of flexible pressure sensors fabricated by photolithography, self-assembly, bulk filling, and nanofibrous structures is superior to sensors fabricated by other methods. However, this is not absolute. The performance of the device is not only related to the microstructure of the force-sensitive interface but also to the device structure of the sensor (Table 2), device materials, detection methods, and other factors. Moreover, the existing preparation method has a guiding effect on the sensing type of the flexible pressure sensor. For example, the current signal such as contact current or tunneling current will be generated between the filling particles after the device is subjected to external forces by the bulk phase filling method, which makes the devices fabricated by the bulk filling method flexible piezoresistive sensors.

**Figure 5:**
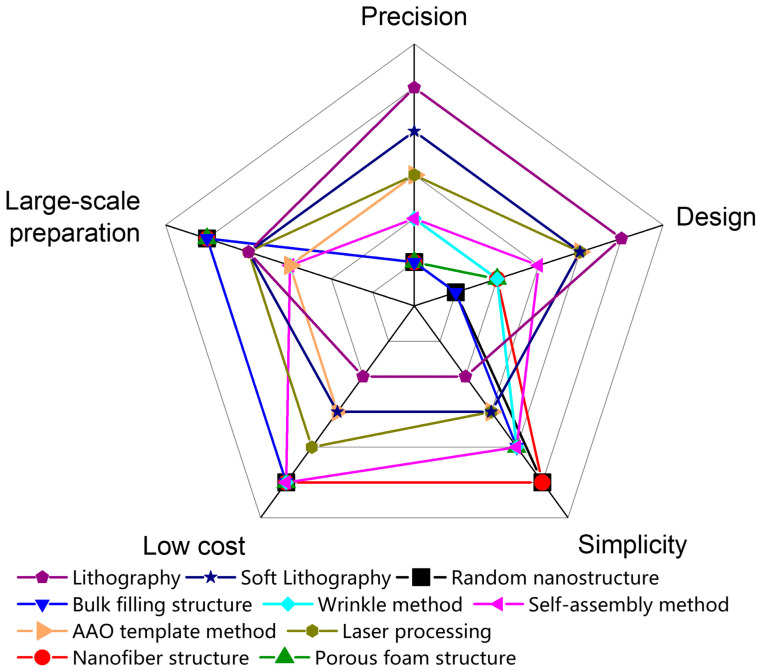
Comparison between the preparation methods of force-sensitive interface microstructures in flexible pressure sensors.

Figure 5 compares the performance of various force-sensitive interface microstructure preparation methods in terms of precision, design, simplicity, low cost, and large-area fabrication. For example, photolithography can design the required microstructures and prepare the microstructures with high precision, but it requires expensive specialized equipment and undergoes more complicated procedures. The preparation of random microstructures has low requirements on templates (animals, plants, sandpaper, etc.), which is easy to produce and has a low production cost, but the precision of the obtained microstructure is low due to the randomness of the microstructure. Generally, researchers can freely choose a suitable microstructure preparation method according to Figure 5.

## 6. Application

### 6.1. Human Health Monitoring

Flexible pressure sensors have broad application prospects in human health and sports monitoring. Minor deformations caused by physiological activities such as blood pulse and breathing, as well as large deformations related to body movement, can be easily detected by the pressure sensor.

#### 6.1.1. Physiological Activity Signal Detection

**Figure 6:**
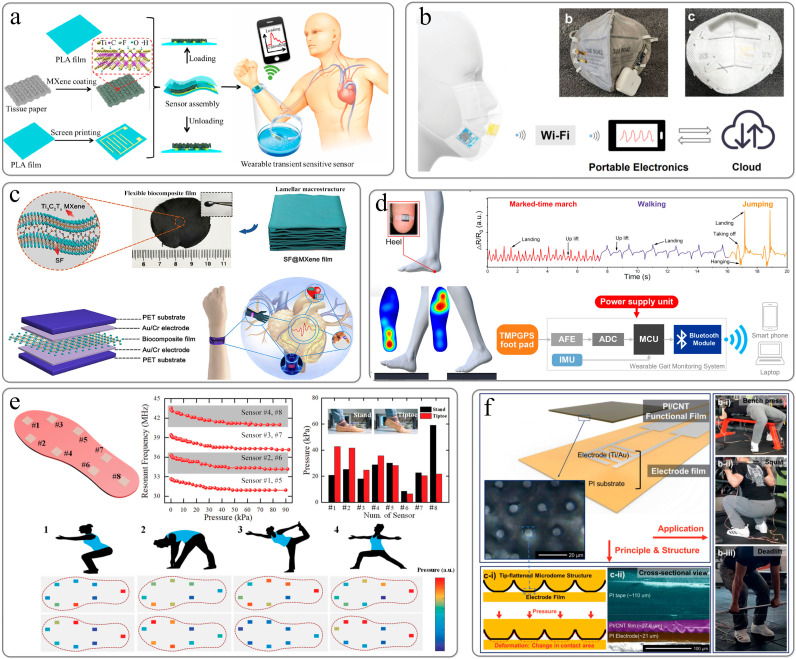
Flexible pressure sensor for human health detection. (**a**) Pulse vibration. Reprinted with permission from reference [135]. Copyright © 2019, American Chemical Society. (**b**) Breath. Reprinted with permission from reference [137]. Copyright © 2021, American Chemical Society. (**c**) Cardiovascular health. Reprinted with permission from reference [92]. Copyright © 2020 Elsevier Ltd. (**d**) Walking posture. Reprinted with permission from reference [138]. Copyright © 2020, American Chemical Society. (**e**) Foot pressure. Reprinted with permission from reference [139]. Copyright © 2019 WILEY-VCH Verlag GmbH & Co., KGaA, Weinheim. (**f**) Body movement. Reprinted with permission from reference [140]. Copyright © 2021 Wiley-VCH GmbH.

Pulse detection and respiratory rate monitoring are essential for the diagnosis of many diseases, such as cardiopulmonary problems, sleep disorders, and asthma. Placing the sensor on the fingertips, wrist, or carotid artery can measure the pulse beat, and placing the sensor under the chest or nose can be used to measure breathing-related deformation. Li et al. designed an ultra-thin flexible piezoresistive sensor with high sensitivity and wide detection range based on pressure-sensitive materials with hierarchical nanonetwork structure and nanonetwork electrodes [133]. The superior sensing performance with the ultra-thin feature and nanonetwork structure provides a high degree of skin fit for the pressure sensor. These superior performances lay the foundation for the application of pressure sensors in physiological signal monitoring and pressure spatial distribution detection. Chang et al. used a hybrid nanonetwork combined with biomaterials as the interface conductive layer of the contact resistive pressure sensor [134]. By effectively modulating the interlayer resistance, the piezoresistive response was significantly improved, and good stability was provided. The developed flexible sensor can monitor the wrist pulse wave under the external moderate pressure level in real time. Guo et al. sandwiched porous Mxene-impregnated tissue paper between a polylactic acid (PLA) sheet and a polylactic acid sheet coated with interdigital electrodes to make a highly sensitive, flexible, and degradable pressure sensor [135]. The sensor can be used to predict the patient’s potential health status and as an electronic skin (E-skin) to map tactile stimuli. It has great potential in personal health monitoring, clinical diagnosis and next-generation artificial skin (Figure 6a). Guan et al. proposed a bio-inspired resistive pressure sensor based on gradient porous materials, which can measure pressures from 20 Pa to 1.2 MPa [136]. The sensor has been successfully used to detect wrist and jugular pulse signals due to its rational structural design and fine material property engineering. Yang et al. demonstrated a novel flexible paper-based pressure-sensing platform, which is characterized by Mxene-coated tissue paper (MTP) sandwiched between a polyimide encapsulation layer and printed paper with interdigitated electrodes [137]. The flexible pressure sensor is integrated with the signal processing and wireless communication module on the mask to be used as a remote breathing monitoring system to wirelessly detect various breathing conditions and breathing abnormalities (Figure 6b). Dongyi Wang et al. demonstrated a flexible silk @Ti3C2Tx MXene (SF@MXene) biocomposite membrane with a three-dimensional cross-linked structure, using natural silk (SF) as a bridging agent to self-assemble two-dimensional (2D) MXene nanosheets into a continuous wave-shaped-layered macrostructure [92]. The pressure sensor can be applied to human health monitoring, including pulse sensory analysis, sound, swallowing, finger movement, etc. (Figure 6c).

#### 6.1.2. Body Motion Signal Detection 

Flexible pressure sensors also have broad application prospects in attitude detection and other aspects. Real-time monitoring of plantar pressure has important applications in sports injury detection, early diagnosis, and orthopedic treatment [141,142]. Wu et al. demonstrated a low-cost flexible pressure sensor with a positive resistance–pressure response based on laser scribing graphene [138]. The sensor can detect a variety of physiological signals and human actions, and on this basis, a comprehensive gait monitoring system is implemented (Figure 6d). Nie et al. proposed a fabric-based wireless pressure sensor array and developed a smart wireless insole that can map the plantar stress distribution (Figure 6e) [139]. Juan Tao et al. constructed a smart insole system based on a capacitive pressure sensor array [143], which has a vertical poroelastic medium layer for monitoring static and dynamic plantar pressure mapping. Jeong et al. designed an ultra-wide range pressure sensor based on optimizing the microstructure of the polyimide/carbon nanotube (PI/CNT) nanocomposite film [140]. The designed pressure sensor is integrated into the shoes and cushions to monitor the pressure of the hands and feet. The data collected by this system successfully monitored the dynamic changes of body pressure signals (heartbeat, pull-ups, and gait cycle) and analyzed the balance of body movement during weightlifting through Pearson correlation coefficient (PCC) (Figure 6f).

### 6.2. Intelligent Robot

**Figure 7:**
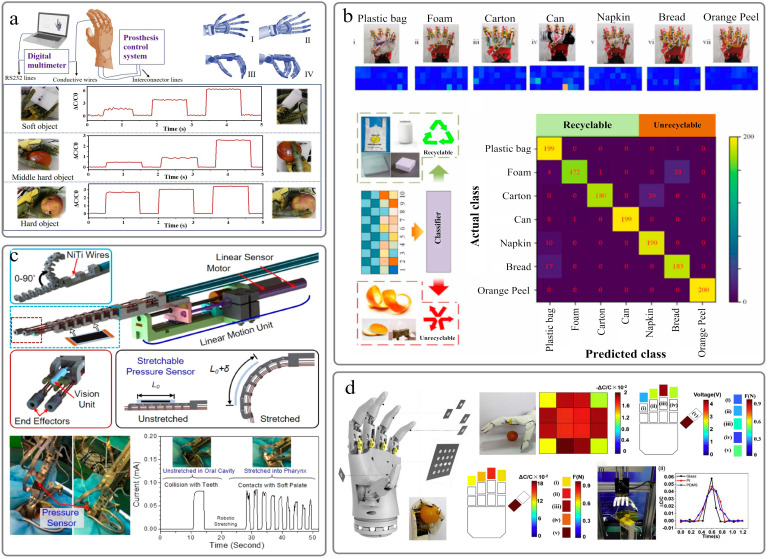
Flexible pressure sensor for smart robot. (**a**) Object recognition. Reprinted with permission from reference [73]. Copyright © 2020 Elsevier Ltd. (**b**) Recognition of object size and shape. Reproduced with permission from reference [144]. Copyright 2020, American Association for the Advancement of Science. (**c**) Collision detection. Reprinted with permission from reference [146]. Copyright © 2019, American Chemical Society. (**d**) Bionic hand. Reproduced with permission from reference [93]. Copyright © 2019 Elsevier Ltd. All rights reserved.

Xiong et al. [73] fabricated a flexible high-sensitivity capacitive pressure sensor based on convex microarray flexible electrodes and an ultra-thin dielectric layer. Due to its excellent comprehensive performance, capacitive sensors have successfully demonstrated their great potential in monitoring physiological signals and robot gripping movements (Figure 7a). Li et al. integrated a quadruple tactile sensor into the manipulator to realize accurate object recognition through grasping [144]. This quadruple tactile sensor consists of a multilayered microstructure inspired by the skin. By combining tactile sensing information and machine learning, smart hands can accurately recognize different shapes, sizes, and materials in various objects (Figure 7b). Jinwon Oh [145] chemically grafted polypyrrole on the surface of porous PDMS elastomer to prepare a piezoresistive pressure sensor. The sensor is installed on the robot pliers to measure the pressure distribution when handling different objects. Chang [146] published a stretchable pressure sensor that consists of reduced graphene oxide (rGO) electrodes with a bionic topology to improve robot collision detection (Figure 7c). Zhang [93] proposed a sensor array that uses a cross-grid liquid metal layer as an electrode and a microstructured dielectric layer as an electrode. It can be used as an electronic skin sensor in capacitive mode or triboelectric nanogenerator (TENG) mode. The dual-mode sensor array is integrated into the palm and fingertips of the bionic hand to verify its application as a robotic electronic skin. Due to the low overall thickness and softness, the array can be conformally attached to the surface of the bionic hand (Figure 7d).

### 6.3. Metaverse

**Figure 8:**
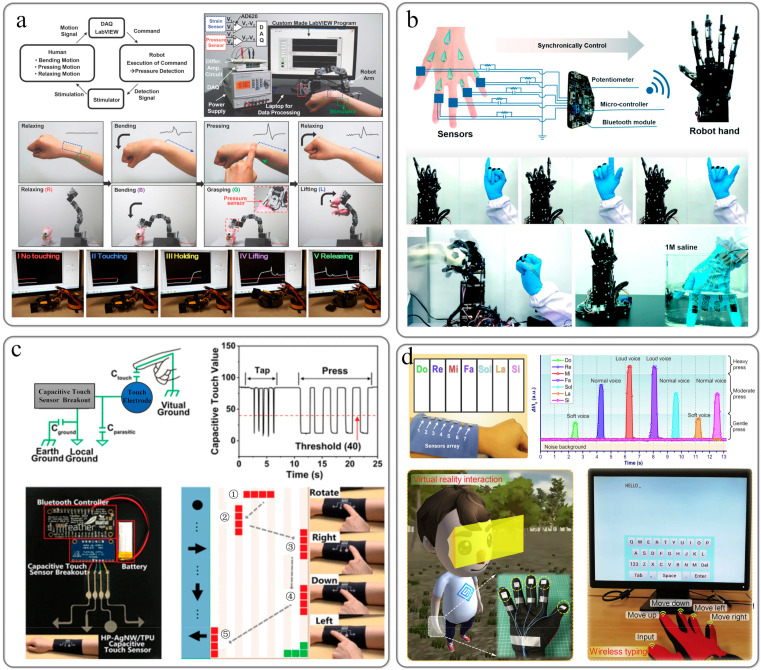
Flexible pressure sensor for Metaverse. (**a**) Obtain human physiological signals through flexible sensors and assist manipulator operation. Reprinted with permission from reference [147]. Copyright © 2014 WILEY-VCH Verlag GmbH & Co., KGaA, Weinheim. (**b**) Real-time control of manipulators through sensors. Reprinted with permission from reference [148]. Copyright 2019, Royal Society of Chemistry. (**c**) Control the movement of Tetris in the game through the function keys on the flexible sensor sleeve. Reprinted with permission from reference [149]. Copyright © 2020, American Chemical Society. (**d**) VR interaction through flexible pressure sensors. Reproduced with permission from reference [150]. Copyright © 2019 Elsevier Ltd. All rights reserved.

Thanks to the development of AR and VR technology and related equipment, wearable devices will become the core technology to enhance the immersive interactive experience of the meta universe. Human–computer interaction technology can provide a more natural and intuitive display method to communicate with computers or robots. Sumin Lim [147] created a transparent and retractable intelligent human–computer interaction system composed of wearable mechanical sensors and stimulators. The system was used to demonstrate the control of various motions of the robot arm and the feedback stimulation upon the successful execution of commands. Yichuan Wu demonstrated the strain and pressure-sensing capabilities of piezoresistive strain sensors [94]. This highly sensitive strain sensor has been demonstrated in human–machine interface demonstrations, which can not only measure human pulse, finger pressure, and finger-bending physiological signals but also assist the robotic arm in grasping and releasing operations (Figure 8a). 

Zhang et al. combined the entangled carbon nanotube network, the combined PPy-PDA-PFDS polymer layer, and the textile substrate to form a sensor through a layered construction strategy [148]. Based on smart gloves equipped with sensor arrays, a human–computer interaction system has been developed that integrates the array glove with a custom data acquisition system and manipulator (Figure 8b). Zhou et al. [149] proposed a gas-permeable, ultra-thin, and stretchable electrode and fabricated a flexible pressure sensor through self-assembly of porous substrates and conductive nanostructures. A piece of HP-AgNW/TPU film is integrated on the fabric sleeve, and laser cutting is used to pattern it into four touch sensors/keys. The four keys are assigned four functions: left, down, rotation, and right. It can be used as a wireless human–machine interface to play Tetris on the computer monitor by putting the sleeve on the arm. When you press the rotate, left, right, and down buttons on the sleeve, the tiles in the game will produce corresponding real-time responses (Figure 8c). Liao et al. proposed a heterogeneous contact microstructure (HeCM) to fabricate a tactile sensor by using silver nanowire @ polyurethane scaffold combined with a layered carbon fabric [150]. A wearable three-dimensional (3D) tactile panel was assembled with seven HeCM tactile sensors. With the help of simple analog-to-digital conversion (ADC) circuits, wearable 3D haptic panels can transform virtual thoughts into realistic sounds. By assembling gloves with five HeCM tactile sensors, a force feedback data glove that can sense fingertip pressure was made for the free control of virtual navigation. In addition, before experiencing the immersive virtual environment, data gloves were used to control the cursor position for wireless-type training.

## 7. Conclusions and Outlook

Flexible pressure sensors are the core components of existing smart electronic devices, playing an indispensable role in smart homes, smart wearable devices, robots, and human–computer interaction. The actual application of flexible pressure sensors requires a good sensing performance. Therefore, many experimental studies are now carried out, from material selection and optimization, force-sensitive interface, and device structure design to research and analysis. However, owing to considerable time and effort needed by the development of new materials, more attention should be paid to the design of the force-sensitive interface and device structure, which can possibly improve the performance of the flexible sensor device in a short time. This article focuses on the regulation of the flexible pressure sensor performance by the micro-nanostructure of the force-sensitive interface of the device and first briefly introduces the existing materials and principles of the device. Then, the microstructures introduced into the force-sensitive interface of the device are classified into periodic/quasi-periodic force-sensitive interfaces and disordered force-sensitive interfaces according to whether they are ordered or not. Periodic/quasi-periodical force-sensitive interface control methods for flexible pressure sensors include lithography, soft lithography, laser printing, AAO template, self-assembly, and 3D printing. The control methods of a disordered force-sensitive interface include bulk filling, foam structure, nanofiber structure, random structure, and other methods. Subsequently, we expand from the force-sensitive interface to the entire flexible pressure sensor device and summarize the existing device structure into a single-sided force-sensitive structure, an interlocking structure, a random multilevel structure, and a structure with a force–electric coupling spacer layer. We also discuss and analyze the existing experimental results. Finally, we display the application of existing flexible pressure sensors in the fields of human health detection, robotics, and metauniverse, indicating huge application potential in future smart electronic devices.

Looking forward to the future, although great progress has been made in regulating the performance of flexible pressure sensors through the design of the force-sensitive interface structure, there are still certain challenges in the preparation of microstructures, device performance, and practical applications of existing flexible pressure sensors:

1. The existing microstructure preparation methods are diverse, including ordered structure lithography, soft lithography, random microstructure templates of disordered microstructures, etc. These methods have their own advantages and disadvantages. For instance, lithography, soft lithography, and laser printing can prepare ordered structures, but they all require specific equipment or a predesign of microstructures, which requires a lot of time and high cost. The preparation of random structures can be directly copied by sandpaper or plant leaves, but the resulting microstructure depends entirely on the template used. Therefore, it is urgent to develop a convenient, fast, and inexpensive preparation method that can modify the microstructure according to one’s own needs.
2. The existing experimental results are basically derived from the flexible pressure sensor manually packaged by laboratory researchers. Therefore, it is unavoidable to apply a certain pre-pressure to the device during packaging, and there are human errors in the manual packaging, leading to poor consistency and unstable sensing performance in actual production applications. Therefore, it is particularly important to develop a device packaging method that guarantees device sensitivity without manual packaging.
3. In order to compare the sensing performance of flexible pressure sensors, the researchers defined a series of performance indicators, including sensitivity, linearity, response time, etc. However, most of the existing experimental studies only pursue high device sensitivity while ignoring the importance of other performance parameters. The flexible pressure sensor device used in the future should be fabricated not only with high sensitivity in a large range and high linearity but also with a fast and stable response.

## Figures and Tables

**Table 1:** Summary of some flexible pressure sensors with different force-sensitive interface microstructures and their performance indicators.

Force-Sensitive Interface Microstructure	Preparation Method		Main Sensing Type	Microstructure Type	Active Materials	Sensitivity	Detection Range [kPa]	LOD [mg]	Response/ Recovery [ms]	Cyclic Stability	Refs
Regular microstructures for force-sensitive interface	Periodic micro-nanostructures	Lithography and Soft Lithography	Resistive Capacitive	Micropillar Micropyramid Micropillar	Graphene v-AuNWs Graphene	10.41 kPa^−1^ (<2.5 kPa) 23 kPa^−1^ (<600 Pa) 3.19 kPa^−1^ (<0.5 kPa)	1.0–32 0–3 0–4	- - 1	19/10 10/10 30/28	>10,000 10,000 500	[49,59,89]
		Laser-induced structures	Resistive	Microcones Microcones	Carbon CNTs	−2.5 kPa^−1^ (0–160 Pa) −0.107 kPa^−1^	0–10 0.005–50	15 -	20 ± 9 200/150	- >10,000	[103,104]
	Quasi-periodic micro-nanostructure	AAO template	Capacitive	Nanopillar Microridge	Cu foil Cu foil	0.35 kPa^−1^ (<2 kPa) 6.583 kPa^−1^ (0–100 Pa)	0.004–25 0–1	40 30	48/60 48/36	3000 10,000	[44,50]
		Wrinkle method	Resistive	wrinkle	GNWs	59.0 kPa^−1^ (0–2 kPa)	0–20	2	6.9/16.1	2000	[39]
		Self-assembly	Resistive Capacitive	Microdome Microdome Microdome	Au Au Au	196 kPa^−1^ (<10 kPa) −15 kPa^−1^ (<100 Pa) 30.2 kPa^−1^ (<130 Pa)	0–100 0–5 0–9	5 40 7	26 <100 25/50	10,000 1000 100,000	[73,107,131]
	3D printing structure		Resistive	-	Au	1.07 kPa^−1^ (<1 kPa)	0–20	-	18	1000	[108]
Irregular microstructures for force-sensitive interface	Bulk filling structure		Resistive	-	Carbon sphere	260.3 kPa^−1^ (<1 kPa)	0–10	-	60/30	5000	[111]
	Porous foam structure		Resistive	-	rGO	0.26 kPa^−1^ (<2 kPa)	0–10	-	-	10,000	[45]
	Nanofiber structure		Resistive	-	MXene	~3.8 kPa^−1^ (0–29 kPa) ~12 kPa^−1^ (29–40 kPa)	0–40	-	26/50	5600	[120]
	Random micro-nanostructure		Resistive Capacitive	Random	Au rGO AgNWs	10 kPa^−1^ (0–400 Pa) 25.1 kPa^−1^ (0–2.6 kPa) 1.54 kPa^−1^ (<1 kPa)	0–7 0–40 0.0006–115	- - -	30 120/80 -	10,000 3000 5000	[48,83,132]

**Table 2:** Summary of some flexible pressure sensors with different device structures and their performance indicators.

Force-Sensitive Interface Microstructure	Device Structure	Main Sensing Type	Microstructure Type	Active Materials	Sensitivity	Detection Range [kPa]	LOD [mg]	Response /Recovery [ms]	Cyclic Stability	Refs
Sensor designs with force-sensitive interface engineering	Single-sided force-sensitive structure	Resistive Capacitive	Microdome -	graphen eMXene GNWs	8.5 kPa^−1^ (0–12 kPa) 151.4 kPa^−1^ (<4.7 kPa) 13.45 kPa^−1^ (0−440 Pa)	0–12 0–15 0–22	10 44 -	40/30 125/104 25/28	10,000 10,000 2000	[40,61,63]
	Interlocked force-sensitive interface	Resistive	Micropillar Nanocone	ZnO NWs PPy	−6.8 kPa^−1^ (0–2 kPa) 268.36 kPa^−1^ (0–200 Pa)	0–13 0–2	6 10	<5 48/56	1000 5000	[13,125]
	Micro-nano-hierarchical structures	Resistive Capacitive	- -	rGO PVA/H_3_PO_4_	1051 kPa^−1^ (50–200 kPa) 3302.9 kPa^−1^ (<10 kPa)	0.01–400 0.00008–360	105 0.8	150/40 9/18	10,000 5000	[38,62]
	Spacer design of force-sensitive interface	Resistive	Wrinkle -	graphene CNTs	28.34 kPa^−1^ (0–3 kPa) 1.91 kPa^−1^	0–10 0–230	22.4 -	- -	6000 74,000	[53,71]

## Data Availability

Not applicable.
